# Food allergy and anaphylaxis – 2044. Component resolved allergen sensitzation profiles in shrimp allergy in the tropics

**DOI:** 10.1186/1939-4551-6-S1-P129

**Published:** 2013-04-23

**Authors:** Irenaeus Paul Chia, Gaik Chin Yap, Bee Wah Lee, Hugo Van Bever, Lynette Shek Pei-Shi, Irvin Francis A Gerez, Genevieve Llanora, Yew Kuang Cheng, Bernard Thong, MA Curotto De Lafaille, Chwee Ying Tang

**Affiliations:** 1 National University of Singapore, Singapore; 2 The Child & Allergy Clinic, Singapore; 3 Pediatrics, NUHS, Singapore; 4 Department of Paediatrics, National University of Singapore, Singapore; 5 National University Health System, University Children's Medical Institute, Department of Paediatrics, Singapore; 6 Tan Tock Seng Hospital, Department of Rheumatology, Allergy and Immunology, Singapore; 7 Singapore Immunology Network, Singapore; 8 Rheumatology, Allergy and Immunology, Tan Tock Seng Hospital, Singapore

## Background

Shellfish allergy is one of the commonest food allergies in the tropics.

## Objectives

To evaluate the sensitization profile to a panel of allergens of dust mite(DM), shrimp and tropomyosin allergens in a cohort of shrimp allergy (SA) patients.

## Methods

Serum allergen-specific IgE of 105 subjects were quantified using ImmunoCAP and ImmunoCAP ISAC biochips. The subjects were classified based on a convincing clinical history and food challenge testing (FC) (dose=70g) to *Penaeus monodon* and *Litopenaeus vannamei*. **Group1A**: Either SA with FC positive to either shrimp (n =22) or SA admitted to emergency departments for severe reactions but no FC performed (n=14) (total n=36); and **Group1B**: Reported SA with FC negative (n=31). **Group2**: Shellfish tolerant DM sensitized controls (n= 38).

## Results

All 105 subjects but one were sensitized to at least one of 3 DM tested (*Dermatophagoides pteronyssinus*, *Dermatophagoides farina*, *Blomia tropicalis*), with highest sensitization rates to Blo t 5 followed by Der f 1. DM and shrimp tropomyosin showed high correlation (p<0.001). **Group1A** had higher rates of sensitization to tropomyosins compared to **Group1B** (Der p 10 [33.3%vs9.7%], and Pen m 1 [33.3%vs9.7%], p<0.037); and to **Group2** (Blo t 10 [19.4%vs0%], Pen m 1 [33.3%vs5.3%], Pen I 1 [27.8%vs5.3%], Lit v 1 [22.2%vs5.3%], p<0.05). Sensitization to Lit v 2 were higher in **Group1A **(22.2%) compared to **Group1B **(6.5%) and **Group2** (5.2%) (p<0.093). The sensitization rates to Lit v 3, 4 were low (<10%). A positive test for a combination of shrimp (ImmunoCAP f24)+any tropomyosin+any shrimp allergens gave the highest sensitivity(81.8%) to distinguish FC positive from negative subjects but had a low specificity of 24.1%. The specificity was highest (93.1%) when using a positive test for Der p 10 or any shrimp tropomyosin, but sensitivity was low (31.8%).

## Conclusions

Tropomyosins are highly cross reactive across species and is significantly associated with SA in the tropics. ISAC Immunocap improved the accuracy to detect FC+ve SA.

